# Laws of Transmission of Resemblance from Parents of Children

**Published:** 1873-12

**Authors:** John Stocktonhough


### ARTICLE IX.

### *The Laws of Transmission of Resemblance from Parents to their Children.*

BY JOHN STOCKTONHOUGH, M. D.

The fact of males resembling their mothers and maternal grandfathers, and females their fathers and paternal grandmothers, was well known to most ancient philosophers and physicians, and many were the reasons given for this condition of resemblances, though none seem to have gone beyond atavism, until in the present day reversion has taken its place. All terms hitherto used as expressive of this condition are without meaning in regard to the proximate cause. The writer will give below what he considers to be the proximate cause, knowing of no other satisfactory explanation as having been given, and offering this as original.

*Males* are begotten from nature ovules (eggs,) and females from immature ovules, hence the *ovule* from which a male is derived is (for a certain length of time, probably from three to seven days) *longer under the sole influence of the mother* than the ovule from which a female is derived, and as the period beginning fecundation and ending with the extrusion of the ovum from the Graafian follicle is claimed by some as that in which reseemblance and hereditary predisposition are impressed, we are led to believe that the sexual differentiation in the resemblance of sons and daughters to their parents is principally due to the difference in the time in which the ovule is under the sole influence of the mother, modified, perhaps, in some degree by the dynamic difference in the ability of the male element to fecundate a mature or an immature ovule, the latter being the more difficult. This, then, appears to be the cause of the embryo, and variations in the condition of the mother during gestation.

Recapitulation :—

1. In general, children of both sexes resemble their mother more than their father in physiognomy, habits, constitution, and temperament.

2. Usually boys resemble their mother more than their father in physiognomy, habits, constitution, and temperament. In the same relationship girls resemble their father more than their mother.

3. As to whether there is any constant relationship between the physiognomical resemblance and a predisposition to the diseases of the person resembled, it is very difficult to decide from the data at hand, but it would appear from the new facts in which any observations were made in this direction, that there was a larger percentage of cases in which inherited diseases were exhibited where there was no resemblance than where there was such physiognomical similitude. In other words, children have resembled one parent in general physiognomy, while they have inherited the constitutional peculiarities and diseases of the other, more frequently than where they have derived both these conditions from (one) the same parent.

This view of the matter is supported by the following cases already recited in this article :—

Mr. Sedgwick's case of icthyosis, where a girl inherited "the disease from her mother," while "she inherited the features of her father."

Dr. Moreau asserts that personal resemblances and cerebral disorder may be transmitted by either parent, but never by the same.

Dr. James Webster assures us that insanity is more frequently transmitted by mothers to their female offspring than to their male children. And Dr. Theophilus Thompson has shown the same fact to be true of the transmission of pulmonary consumption.

The facts are too few to warrant us in defining this as a law, yet I know of no facts of theories to the contrary. I am under the impression, however, that physicians have usually regarded a physiognomical resemblance as evidence of greater liability to the hereditary disease of the person resembled, yet it is quite possible I may be mistaken in this view of the matter.

In general, then, hereditary and acquired diseases and defects are more likely to be transmitted to offspring of the sex in which they originated, and thereafter to be subject to the principle of sexual limitation either directly from parent to child, or by interrupted or atavic descent, from grandparent to grandchild.

Though sons are usually best able to follow the avocation of their fathers, it is undoubtedly true that men inherit the genius, talent, and intellectual excellence and mortality of their mother or their mother's father, while daughters inherit the same qualities from their father or paternal grandmother.

*Females more frequently transmit* hereditary diseases and defects than males, though they *less frequently exhibit* them. Males less frequently transmit, and more frequently exhibit, inherited diseases and defects.

I have already, in my last paper called attention to, and offered an explanation of, the phenomena stated in the last conclusion (4), and may repeat it here:—

The ovule (egg) from which a male is derived being for a longer time under the sole influences of the mother (before fecundation, and longer in utero-gestation) than the egg from which a female is derived, acquires more of her physical constitution and peculiarities, resembles her more, or inherits more of her physical defects and tendencies, and this ovule is fecundated by a weaker element on the father's part than his female offspring; while the ovule (egg) from which a female is derived is a shorter time under the sole influence of the mother, being impregnated earlier in its course of development (being less mature,) and besides this it requires the highest power of the male element to communicate the imprègnating influence to it. Hence we have less hereditary disease *exhibited* in the female, yet she may *transmit* with greater frequency and facility than the male, though it may not have developed in her.

The reason that females do not exhibit hereditary disease as frequently as males, is because of a *higher degree of vitality* in them, which gives them greater power to restrain the appearance of the predisposition, and an *inferior degree of developmental evolution*, retaining in their constitution as germs, what in men become fully developed diseases and defects.—*Medical Recorder*.

